# Climate Change and Its Impact on the Yield of Major Food Crops: Evidence from Pakistan

**DOI:** 10.3390/foods6060039

**Published:** 2017-05-24

**Authors:** Sajjad Ali, Ying Liu, Muhammad Ishaq, Tariq Shah, Abdullah, Aasir Ilyas, Izhar Ud Din

**Affiliations:** 1 College of Economics and Management, Huazhong Agricultural University, Wuhan 430070, China; sajjad@webmail.hzau.edu.cn (S.A.); ishaqecon@gmail.com (M.I.); abdeco@webmail.hzau.edu.cn (A.); aasirilyas@yahoo.com (A.I.); 2 Hubei Collaborative Innovation Center for Grain Industry, Yangtze University, Jingzhou 434025, China; 3 Department of Economics & Development Studies, University of Swat, Khyber Pakhtunkhwa 19130, Pakistan; shah6833@yahoo.com; 4 College of Public Administration, Huazhong Agricultural University, Wuhan 430070, China; committedizhar@webmail.hzau.edu.cn

**Keywords:** Pakistan, climate change, yield, major food crops, food security, agricultural development

## Abstract

Pakistan is vulnerable to climate change, and extreme climatic conditions are threatening food security. This study examines the effects of climate change (e.g., maximum temperature, minimum temperature, rainfall, relative humidity, and the sunshine) on the major crops of Pakistan (e.g., wheat, rice, maize, and sugarcane). The methods of feasible generalized least square (FGLS) and heteroscedasticity and autocorrelation (HAC) consistent standard error were employed using time series data for the period 1989 to 2015. The results of the study reveal that maximum temperature adversely affects wheat production, while the effect of minimum temperature is positive and significant for all crops. Rainfall effect towards the yield of a selected crop is negative, except for wheat. To cope with and mitigate the adverse effects of climate change, there is a need for the development of heat- and drought-resistant high-yielding varieties to ensure food security in the country.

## 1. Introduction

Change in climate is mainly attributed to the unabated increase in greenhouse gases, including fluorinated gases, carbon dioxide, methane, and nitrous oxide, whichbring changes in rain pattern, temperature, and negative effects on water and land resources, floods, and droughts. Climate change is considered to be a global phenomenon; however, its impacts are more widely felt in the developing countries, due to their greater vulnerabilities and lesser ability to mitigate the effects of climate change. Because most developing nations—including Pakistan—are agriculture-based economies, their agricultural sector is affected the most due to direct exposure to nature. Therefore, the major impact of climate change is on agricultural production due to changes in rain pattern, temperature, floods, droughts, and negative effects on water and land resources [1,2]. In the developing states (such as Asia and Africa), the latest work has progressively considered the impacts of climate change on agricultural production [1,2].

Food security and water availability are highly vulnerable to the rapidly changing climate. During the summer season, most of the climate models expected that rainfall will increase [3,4]. The Himalayan glaciers (75%) are melting, and will disappear by 2035. The reduction or escalation in the intensity may result in droughts and floods, respectively [5]. Climate change will affect crop productivity, and can thus cause food security problems [6,7,8]. It has been expected that global warming will increase yields due to “fertilizer effect”, but will influence poor farmers negatively. For example, countries closer to the equator will have reduced production due to global warming [9]. African countries will experience extreme droughts and a further shortage of food. If climate change affects the productivity of the agriculture sector in the lower-income countries of Asia or Africa, a large number of people will be at risk, and the problem of food insecurity will increase. Climate change is the main driver of food security in the developing world, because it affects the productivity of the agriculture sector, its stability, and other components of the food system, including storage, access, and utilization [10].

Generally, climate change has minor effects on global food production, but these effects are unevenly distributed geographically. Most of the losses are suffered in lower-income countries, such as those in arid and sub-humid South Asia and Africa. These regions are engaged in subsistence agriculture, and are not technically sound or financially strong enough to abate the negative impacts of climate change. Moreover, they have almost no potential for adaptation [1]. This adversely affects the people in these countries because of their dependence on agriculture for their livelihood. According to estimates of the Food and Agriculture Organization (FAO), the global number of undernourished people is 795 million. This shows a decline of nearly 200 million over the last 20 years [11]. South Asia and Sub-Saharan Africa constitute most of the world’s hungry population [12]; the most vulnerable region to climate change is South Asia [13]. In South Asia, more than 70% of the people (approximately 1.1 billion) live in rural regions dominated by agriculture, and almost 75% of these people are poor [14]. More importantly, nearly 18% of the region’s GDP is comprised of agriculture, and the industry employs more than 50% of the population [14]. In addition, climate change can pose threats to agriculture and food security by changing the spatial and temporal distribution of rainfall, water availability, land, capital, biodiversity, and terrestrial resources. Due to changes in agricultural production, it could potentially cause food uncertainty for 9 billion people by 2050. Research shows that regional and global water requirements can be altered due to climate change and can trigger a shortage of water for agricultural purposes.

Studies reveal that increasing temperature and the changing pattern of rainfall have a substantial impact on food production [8,15,16]. A recent study anticipates that the wheat production of South Asia will decline by 50% by 2050—equal to almost 7% of the global crop production [17].

The Peterson Institute states that agricultural production in developing countries will further fall between 10% to 25% and global warming will decrease the agricultural capacity of India by 40% if it continues unabated [18]. Hence, climate change causes serious threats to food security [6,19,20,21], negative impacts on productivity of different crops, the food supply [22,23], and the cost of adoption of climate change is high [24].

Therefore, food security has remained a prominent policy of Pakistan’s government. Wheat, rice, and maize are the main food crops, and sugar cane is the main cash crop of Pakistan. Therefore, food security policy mainly focuses on the production of these crops. In light of the above deliberations, this study aims to examine the nexus between climate change (maximum temperature, minimum temperature, rainfall, humidity, and sunshine) and the yield of major crops including wheat, rice, maize, and sugarcane.

### Review of Literature

Study of global climate models (GCMs) projects reveal remarkable alterations to regional and globally averaged precipitation and air temperature, and these alterations will probably have associated impacts on groundwater recharge. A researcher predicts that the rainfall pattern, river flows, and sea levels all over the world will be affected due to climate change over the next century [25]. Remarkable changes in the climate system may severely affect the agricultural yield over the next hundred years.

The increase in climate change is recognized as a global anomaly with potentially long-lasting implications, corresponding with more frequent extreme weather episodes [26]. Such climatic alterations are assumed to affect people residing in agricultural communities of developing countries [27]. Only 10% of annual global CO_2_ is emitted by developing countries, and yet they are the most susceptible to climate change [27]. The large population in South Asian countries is dependent on rural economies based on agriculture; hence, these areas are especially affected by climate changes. This causes serious threats to their social, economic, and ecological systems [28]. The World Bank’s South Asia Climate Change Strategy announced that the poorest people in the region will be affected the most by climate change due to limited assets, unfavorable geography, and a greater dependence on climate-sensitive sources of income. Extreme weather events at high frequency in the region in recent years (e.g., flash floods in Pakistan and India) are thought to be directly associated with climate change and are likely to keep the poor in a perennial poverty trap [29].

It is expected that the majority of the impact of climate change will affect the agriculture sector the most due to its vulnerability [30]. The variability in climate change presents a major challenge to agricultural production and rural livelihoods, as it affects approximately 2.5 billion people who are partially or completely dependent on agriculture. The agricultural sector is highly dependent on alterations in climatic conditions, thus making it a dangerous activity [31,32,33,34].

Global agricultural production is challenged by climate change [35]. Climate change affects different crops and regions differently, but it is generally expected that agricultural productivity will decline [36]. In fact, some decline can already be seen. According to one calculation, climate change resulted in the reduction of global maize yield by 3.8% between 1980 and 2010 [37]. Farmers were not threatened evenly compared to crops, but they were the most vulnerable group to climate change [38]. Economic losses from extreme weather conditions are increasing due to climate change [39]. It is projected that average temperature will increase and patterns of rainfall will change. Consequently, it is likely that more intense events and floods will occur in the coming decades. This will greatly affect the community in terms of rescue operations, loss of human life, damage to assets, and disruption of business [40]. It is difficult for the government to pay for the damages. Crop productivity is affected by climate change, with serious implications for food security [6]. Global warming has been hypothesized to boost yields, as increasing atmospheric carbon acts as a fertilizer effect, but the impacts are probably negative overall for poor countries. For instance, there will be a reduction of food production in countries near to the equator due to global warming [9]. There will be extended droughts and food shortages in African countries. There will be more poverty and other social troubles in Pacific Islands and Indonesia, as these countries are more dependent on imports. A recent International Water Management Institute (IWMI) study forecasts that wheat production in South Asia will decline by 50% by 2050 [17].

The agriculture sector is highly dependent on alterations in climatic conditions, thus making it a dangerous activity. Climate variability is a major source of risk for agriculture and food systems. The increasing severity and frequency of extreme weather have extensively flawed agriculture [31]. Farmers are regularly facing natural disasters, capricious rainfall, and pests. For example, farmers are encountered with heavy rains, floods, pests and diseases [32], droughts and market price variations [33]. According to a report on production, financial, marketing, legal, environmental, and human resources are significant sources of risk factors in agriculture [34].There are five major risk factors, as follows: production risks linked with changes in crop yields and livestock from many sources (i.e., unpredictable weather conditions, disease incidence, and pests). Secondly, there are financial risks, such as a farmer’s capacity to pay their bills to sustain farming and avoid liquidation. Thirdly, marketing risks, which involve variations in the prices of agricultural products. Fourth, there are legal and environmental risks, and finally there are narrow human resources (i.e., a lack of family members to play the role of labor and farm management). As a consequence, there is a negative impact on production, leading to huge production losses. It is therefore important for farmers to perceive and regulate production risks appropriately [41].

The results of the study show that more than 50% of farmers were risk hostile in nature, and they had high perceptions about floods. In case of economic loss, flooding is the most calamitous natural disaster. All studies revealed that farmers were the most affected in terms of damages to crops, water contamination, irrigation systems, livestock, and other agricultural operations. Moreover, losses in farm yield and security increased due to the negative impacts of floods on agricultural systems. The same results were obtained by [42,43]. Due to these tremendous losses to agriculture production in 2010, 2011, and 2014, farmers considered floods and heavy rains to carry much greater risk than other natural disasters. This high risk-perception of farmers resulted in a high-risk-averse attitude in farmers. The results for risk aversion were related with the findings of [33]. They announced that the majority of farmers in their studies were risk averse in nature. Education was crucial among socioeconomic factors because it affected the risk aversion behavior of farmers. Educated farmers may be capable of successful perceptions and adoption to minimize or avoid risk due to their understanding and knowledge. These findings for the link of education with risk aversion are correlated with the following findings [44]. According to their study, most of the educated farmers in the Philippines were risk averse in nature compared to illiterate farmers. The same findings for education and risk attitudes of farmers were also reported in [45]. However, some have reported a contrary relationship [46]. They described that as their education augmented, farmers were less risk averse in nature. In terms of experience, our findings showed that there is more risk aversion in highly experienced farmers as compared to those having less experience. Experienced farmers are less likely to confront the natural disasters because of their indigenous knowledge about prevailing environment and weather conditions. These findings are also related to the findings of others (e.g., [44]). Their results illustrated that there is more risk aversion in highly experienced farmers as compared to less-experienced farmers.

It was also revealed that there was more risk aversion in farmers with greater high risk perception as compared to those with lower perception. Risk perception is a very crucial signal in the disasters literature. Individual and community responses to natural disasters can be illustrated, and a positive link is found between public response and adaptation to natural hazards [47]. This means that when the risk perception of farmers directly affects the risk aversion of farmers, they will adopt risk minimizing strategies. For example, farmers having high risk perception of floods prefer to cultivate off-land and to practice diversification as agricultural flood-risk management tools [32]. In the same way, farmers may use diversification in income, precautionary savings, and diversification in crops and many other farm risk management tools in before and after disaster situations.

Large farmers have more land and greater diversification of income and crops. Therefore, the dummy for the farmer category reveals that large subsistence farmers have less risk aversion as compared to the small subsistence farmers. Hence, farmers’ socioeconomic factors and other disaster-related factors play basic roles in determining their risk attitude. After the 2005 earthquake and major floods in 2010, Pakistan still has poor disaster management, preparedness mitigation, and institutionalized coping strategies. It is significant that disaster risk reduction and preparedness should be a national priority. Moreover, it is obligatory that disaster-prone areas should be included in government support programs. The interests of the farming community can be secured by such policies initiated by government [48].

Agriculture and food security can be affected by change in climate for many reasons, such as the rainfall distribution and the availability of capital, water, biodiversity, land, and global resources. This may increase doubts on the food chain ranging from farm to fork and result in trade dynamics, and eventually affect the global economy, food security, and the capability to nourish 9 billion people by 2050. Modeling by International Institute for Applied System Analysis (IIASA) showed that irrigation requirements at the regional or global level may affect the climatic conditions, agricultural water withdrawals, and future socioeconomics [49]. It is also observed that this irrigation requirement may increase by up to 45% by 2080. Even with upgrades in the irrigation system, overall there may be 20% water withdrawals increases. Global irrigation supplement with change in climate will boost by 20% above the reference base case scenario (exclusive of climate change). The reproduction shows that climate change largely affects irrigation water requirements globally similar to the increase in irrigation due to socioeconomic development. Climate change has little impact on global food production, but its geographic distribution is uneven. For example, there are more losses in Africa and South Asia representing arid and sub-humid areas [50] and specifically in poor countries with less ability to adapt to climate change [1].

Dry hot summers and freezing cold winters prevail in Pakistan. The geographical characteristics of the country are divergent; the country has high mountains (mountainous systems in the north, center, north-west, and south-west), plateaus in the center, and plains, deserts, and a lengthy coastline in the south west. Each geographic location is characterized by different climatic conditions; some regions are very cold, and some are very hot, while some of them remain moderate all year. However, the historical data shows that there is less precipitation in this region as compared to adjacent areas. The country has a rich river water system, which is the major source fulfilling agricultural water requirement [51]. The role of agriculture is very important in ensuring food security and decreasing poverty. Temperature increase can affect agriculture through its effect on cropping seasons, the increase in irrigation, the increase in evapotranspiration, and the increasing effect of heat stress on crops. Short duration crop varieties, cultivating, and modification in crop sowing time may reduce the negative impact of the aforementioned climatic threats [52].

Because of disparate climatic conditions, the susceptibility index of climate change in Pakistan is very high as compared to several countries around the globe. Recently, the country has faced climatic changes including enhanced temperature, variations in precipitation pattern, weather shift, floods, earthquakes, and more. Pakistan—though not a major contributor to emissions that have resulted in creating the climate change situation—has a high vulnerability index. The needs for its adaptation to new changes are very high [51]. Since 2010, the agricultural sector in Pakistan has faced three gigantic floods that had overwhelming effects on the whole economy, and predominantly the agriculture sector. A massive destruction in 2010, 2011, and 2014 to agricultural crops, livestock, forestry, and fisheries occurred due to monsoon floods and also caused damage to key infrastructure such as animal shelters, tube wells, fertilizers, houses, water channels, people, seed stocks, and agricultural equipment. Before the harvesting season of the main crops including wheat, rice, maize, sugarcane, and vegetables, a devastating flood occurred. An approximate loss in production of 13.3 million tons was recorded due to the yield loss of major crops. Standing crops worth 2 million hectares were destroyed, while 1.2 million livestock including poultry were lost to the flood of 2010 [53]. Another gigantic flood came in 2011 in the provinces of Baluchistan and Sindh that severely affected these provinces and also the people living in them in the sense of loss of lives, particularly concerning to agricultural activities and issues. About 80% of the population living in the rural areas of Sindh is reliant upon agricultural activities for their livelihoods, fisheries, crops, forestry, and livestock [54]. The flood ruined standing crops of rice, sugar cane, vegetables and pulses, sorghum, cotton, and livestock, along with heavy losses of lives in 2011. For instance, around 115,500 livestock were killed; about 5 million livestock survived, but they were also obliquely affected through disease and dislocation. The predictable total loss was US$1840.3 million, out of which 89% was direct damage and 11% indirect victims. The maximum damage (about US$1.84 billion) happened in the agricultural sector, predominantly to livestock and fisheries. The total damage of US$3.7 billion has been estimated due to floods in 2011. The total cost of US$2.7 billion has been estimated for revival and rehabilitation [55]. More than 2.5 million people were affected by heavy rains and floods, and 367 persons died in the current floods in September 2014. Furthermore, 250,000 farmers, 129,880 houses, and more than 1 million acres of cultivated land were affected. The cost of recovery and resilience building were estimated at US$439.7 million and US$56.2 million, respectively [56]. These statistics demonstrate that agriculture was the sector most affected by floods in Pakistan.

There is great interest in adaptation, which leads to several definitions of climate adaptation. It is defined by the Intergovernmental Panel on Climate Change (IPCC) as “*The adjustment in natural or human systems in response to actual or expected climatic stimuli or their effects, which moderates harm or exploits beneficial opportunities*”. Others focused on a much broader definition, which is accompanied by any action which improves the situation of society caused by climate change [37]. The previous literature indicates that a certain level of drought and improper use of land might have an impact on the local agriculture, whereas the losses due to disaster can be reduced by the rational management of land use [57]. For example, a long-term land use policy was needed to counter the negative impacts of climate change in Malaysia [58]. Similarly, several ways were found to cope with climate change, such as the use of modern seeds [59], changing plantation dates, and changing the type of farming in South Africa [60]. In China, it was shown that the government tried to adapt to climate changes over the past decade by starting nationwide projects of reforestation, which provides substantial environmental benefits [61].

Efforts to sustain the global food system are suffering from the serious challenge of agricultural vulnerability to climate change. The negative impacts of climate change such as increase in temperature and variation in rainfall are expected to lower the benefits for production of the agricultural sector [62]. The Midwestern US Corn Belt contributes substantially to this system through the production of more than one-third of the world’s supply of corn. The agriculture sector of the US is contributing substantially to greenhouse gas (GHG) emissions, and is vulnerable to change in patterns of weather, diseases, and pests [26].

Adaptation to climate change is taken to reduce the negative impact of climate change. Moreover, adaptation could be classified into spatial (both localized and widespread), and may also be in the form of behavioral, technological, institutional, informational, and financial adaptations [63]. The Kyoto Protocol and IPCC agreed that adaptation has different forms [64]. Adaptation has been explained in terms of vulnerability to climate change. Hence, adaptation to climate change is linked with the perception of what climate change is. This is how individuals or groups have to respond to the change in climate. The perception of an individual depends on her experience and knowledge and the observed impact of climate change. For instance, when there was a change in temperature in 11 African countries, the farmers changed crop varieties, increased conservation of water, and switched to non-farm activities [65]. On the other hand, when there was change in precipitation, they changed planting dates. Similarly in a study conducted for South Africa and Ethiopia including 1800 farmer households, it was observed that the strategies commonly adopted by farmers consist of planting different crops and varieties, cultivating trees, practicing soil conservation, and changing dates of planting and irrigation [60]. However, it was observed that farmers who did not adopt any adaptation strategy revealed that lacking credit, lack of information, and access to land were the major factors which obstructed them from adapting to perceived change in climate [60].

Pakistan is deemed to be one of the highly vulnerable countries to climate change, as reported by [66]. It was ranked 21st by the Global Climate Risk Index (GCRI) in terms of exposure to extreme weather conditions for the period from 1993 to 2012 [66]. Pakistan was listed as the twelfth-most highly exposed country to climate change by the World Bank [67]. The economy of Pakistan is based on agriculture, which contributes 19.8% to the GDP to and employs about 42.3% of the labor force, and is providing livelihood opportunities for approximately 62% of the rural population [68]. Besides its importance for Pakistan, this sector is facing serious challenges from changes in climate, such as rise in temperature, droughts, floods, and losses in yields [67]. Over the last few decades, the effects of climate change have become apparent, with mostly lower-income countries being affected [69]. Pakistan is experiencing extreme climate events including high temperatures, floods, shortage of water, droughts, and increased attack of diseases and pests [70]. The ranking of Pakistan with respect to climate change vulnerability is bad, as it stood at 29th in the list of most vulnerable countries for 2009–2010 and ranked 16th in 2010–2011 by the Global Climate Change Vulnerability Index (CCVI) [71]. The severe floods starting from 2010 to 2014 and droughts ranging from 1999 to 2003 are among the few examples related to climate events of Pakistan. It is expected that climate change will have an adverse effect on the economy of Pakistan, as extreme weather events are already occurring, such as changes in rainfall patterns, droughts, and floods [72]. Pakistan is especially vulnerable to change in climate due to its dependence on natural resources. Thus, appropriate adaptation measures are need [73].

Therefore, looking into the current and the future expected changes in climate, this study is planned to examine the effects of climate change in terms of maximum temperature, minimum temperature, relative humidity, sunshine, and rainfall on the major crops of Pakistan. This study is important, as Pakistan is vulnerable to climate change and the food security situation across the country is not very good.

## 2. Methodology

### 2.1. Climatic Features of Major Food Crops in Pakistan

Pakistan’s economy is based on the agricultural sector because of its contribution to the total income of the country. The agricultural sector serves as a source of livelihood for 42.3 percent of rural inhabitants and contributes 19.8 percent to the gross domestic product (GDP) of the country. The prime objective of the agricultural sector is to ensure food security and reduce poverty by increasing production. The country is extremely vulnerable to climate change due to its geographical location, high population, and low technological resource base. Pakistan suffered economic losses of more than US$15 billion during the floods of 2010. About 300,000 people were displaced, and more than 20 million seriously affected. Pakistan needs US $6–14 billion annually to mitigate the negative impacts of climate change. Some of the gifts of climate change to Pakistan are the floods of 2013, 2011, and 2010, extreme droughts of 1999–2003, and the cyclones in Karachi/Gwadar in 2008 which led to landslides and glacial lake outburst floods (GLOFS) in Northern Pakistan. Climate change poses a serious challenge to Pakistan by threatening its food, water, and energy security owing to a possible change in weather pattern, extreme events such as floods, droughts, and heat waves, and reduces agricultural productivity. About 5000 glaciers in Pakistan are retreating, and their retreat speed is faster than the rest of the world [74].

**Figure 1:**
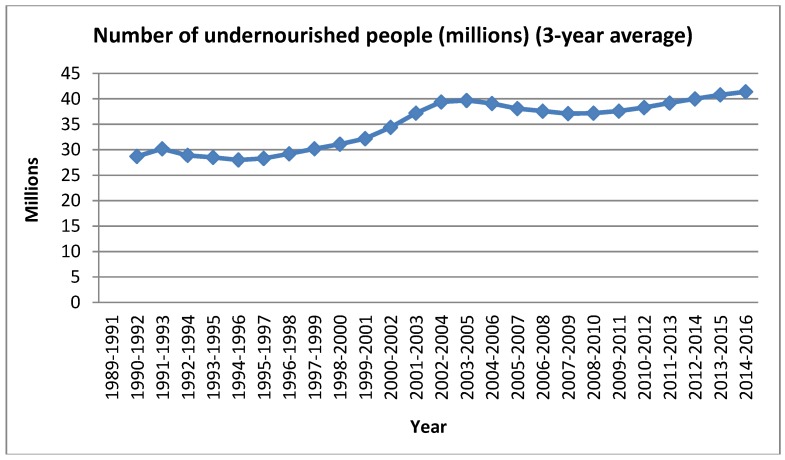
Number of People Undernourished (3-years average).

Pakistan is among the list of countries where almost 65% of the world’s population lives and is suffering from the problem of food insecurity. These countries include Congo, China, India, Indonesia, Pakistan, Ethiopia, and Bangladesh [75]. The worst countries in terms of food insecurity and which are unable to take practical steps to overcome this issue include many Asian and African countries. Moreover, they are unable to meet the millennium development goals and hunger eradication objectives [76]. The number of undernourished people in Pakistan is given in Figure 1.

**Figure 2:**
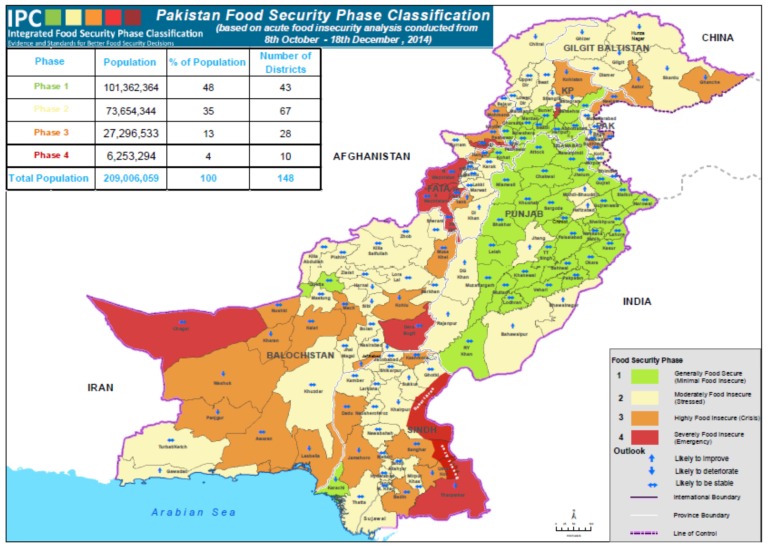
Pakistanfood security phase classification.

**Figure 3:**
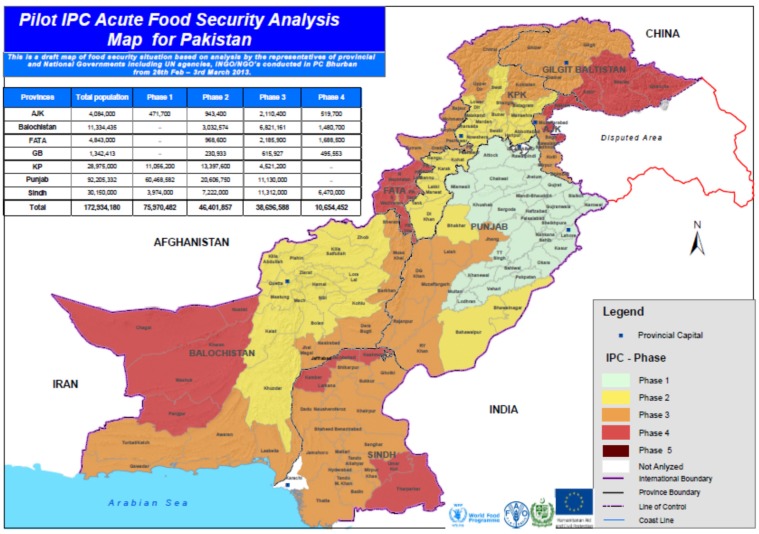
Acute food security analysis map for Pakistan.

The major food crop of Pakistan is wheat. Other important crops are rice, maize, and sugarcane, which contributed to agriculture by a rate of 31.9% in value addition, showing 3.2% growth over the period 2011–2012. Wheat has been considered as one of the important staple foods in Pakistan since the 1960s, and it is a major contributor to GDP. Pakistan is a self-sufficient country, as reported by [77]. Many agricultural commodities are produced by Pakistan. Disregarding the various efforts and investment by the government, it is estimated that nearly 22% of the population is food insecure [76]. The food security situation of Pakistan is given by Figure 2 and Figure 3.

The policy makers of Pakistan mainly focus on wheat, as it is an important staple food. In Pakistan, wheat is an important food crop due to its extensive use as a food in daily life and as a cheaper source for animal feeding. Out of a 22.45 million ha cultivated area, only 6.34 million ha land is irrigated by canal water, and nearly 12.52 million ha is cultivated through tube wells and other sources. There is no availability of water for the remaining 3.59 million ha [55]. The sowing season for wheat in Pakistan is November. About 9045 thousand hectares of land are used to cultivate wheat, while the yield is about 2657 kg [78]. This crop is very important in Pakistan, as consumption per head of wheat is nearly 120 kg. Currently, about 26 MAF (million acre feet) of water is available for cultivation of wheat, which is lower than the required amount by approximately 28.6 percent [79]. Approximately all models predict that climate change would negatively affect the yield of wheat in South Asia. The report of the 4th IPCC revealed that crop yield in south Asia would decrease by percent by 2050 from 1820 m^3^ to 1140 m^3^ from 2001 to 2050. In addition, a decline in the availability of gross water per capita would also be observed. The supply of water is scare in most parts of the country. It is expected that in the near future, the availability of water would decrease sharply, as would the production of agriculture sector.

### 2.2. Data Sources

This study aims to assess the impact of climate change variables, including temperature (maximum and minimum), rainfall, humidity, and sunshine (radiation) on the yield of major food crops (wheat, rice, and maize) and cash crop (sugarcane) grown in Pakistan. This study is based on secondary data covering the period from 1989 to 2015. District-wise yearly data on the yield of the reported crops was collected from various issues of the Agricultural Statistics of Pakistan published annually by the Pakistan Bureau of Statistics [80]. Daily data on climate variables were collected from the Pakistan Meteorological Department for the same period [81]. Climate data included information from10 different weather stations. The daily data on climate variables were transformed accordingto crop season starting from sowing to harvesting dates that vary across the districts to represent the whole of the country.

### 2.3. Model Specification

It is expected that each crop needs certain conditions for growth and survival, such as soil moisture (water), optimum temperature, adequate sunshine, and atmospheric humidity. According to one study, temperature (both maximum and minimum) enhances photosynthesis as it increases and leads to an increase in crop yield [82]. However, it was stated that extremely high temperature negatively affects the process of metabolism in plants, such as protein stability and reactions (enzymatic) in cells, leading to metabolic imbalance [83]. In addition, high temperature also affects photosystem II and RuBisCO function, and causes a reduction in photosynthesis. On the other side, the extremely low temperature may lead to injury of chilling in plants [84]. In monsoon season, intense rainfall can be a serious problem for the farmers. It can lead to soil erosion, washing out of soil surface, and depletion of nutrients in the soil. Devastating flood can destroy crops, leaving no food for farmers to eat and sell, and thus can trigger production and food security problems [85]. Similarly, humidity also plays a crucial role in crop yield. Plants need sufficient humidity for their balanced growth. Humidity that is too low or too high is not beneficial for higher crop yield. When humidity is sufficient, plants can absorb nutrients from the soil and can provide a high yield. Plant–water relationship is directly controlled by humidity and indirectly by the growth of leaves, rates of photosynthesis, pollination, disease occurrence, and lastly, crops yield. Furthermore, dryness in the atmosphere can seriously threaten dry matter production by controlling stomatal leaf water potential [86]. A key determinant of plant growth is sunshine, because it affects the process of photosynthesis. As we know, plants directly depend on sunshine for their healthy growth and development, completion of their life cycle, and most importantly, food preparation. In contrast, excessive sunshine has similar negative effects on crops (e.g., extreme temperature) [87]. Therefore, climate change is expected to have various significant impacts on crop yield.

In light of the above deliberations, this study estimates the following model to meet the objectives of the study. The feasible generalized least square (FGLS) and heteroskedasticity and autocorrelation (HAC) consistent standard error techniques are used, keeping in view the nature of the dependent and independent variables [88].

(1) $$Y_{it}=\alpha_{0}+\alpha_{1}Max_{it}+\alpha_{2}Min_{it}+\alpha_{3}Rain_{it}+\alpha_{4}Hum_{it}+\alpha_{5}Sun_{it}+\alpha_{6}Year_{it}+\varepsilon_{it}$$

where $Y_{it}$ = Yield (kg/Acre) of crop i in year t; $\alpha_{i}$ = Coefficient to be estimated; $Max_{it}$ = Average monthly maximum temperature (°C) during cropping season for crop i in confirmed year t; $Min_{it}$ = Average monthly minimum temperature (°C) during cropping season for crop i in year t; $Rain_{it}$ = Average monthly rainfall (mm) during cropping season for crop i in year t; $Hum_{it}$ = Average monthly humidity (%) during cropping season for crop i in year t; $Sun_{it}$ = Average sunshine (hours per month) during cropping season for crop i in year t; ε = error term.

## 3. Results and Discussion

### 3.1. Descriptive Statistics

The descriptive statistics are calculated in Table 1, to show the basic properties of all the variables in this study. The purpose of this study is to find the mean yield, cropping area, and production for all the major crops. In the case of yield, it was found that among the four crops, the mean yield of sugarcane was the highest. According to this study, the observed mean yield of the four crops in descending order is as follows: sugarcane > maize > wheat > rice. In the case of average production, sugarcane ranked the highest, wheat stood second, maize third, and rice ranked the last. According to the average production area, wheat ranked first, and sugarcane, maize, and rice stood second, third, and fourth place, respectively. In case of climate variables, the highest maximum temperature was noticed in the rice growing season and the lowest maximum temperature was observed in the wheat growing season. In contrast, the highest minimum temperature was observed in the rice growing season, while the lowest minimum temperature was noticed in the wheat growing season. For rainfall, rice received the highest rainfall in growing season; maize and sugarcane were in second and third place, while wheat received the lowest total rainfall. In view of relative humidity, the highest percentage of humidity was detected in the sugarcane growing season, while the lowest value was detected in the maize growing season. Finally, in the case of sunshine, the highest sunshine was observed in the rice growing season and lowest was seen in the wheat season. This study will also determine which climatic variable affect food crops the most severely in Pakistan.

### 3.2. Trend Graph

In addition to examining descriptive statistics, graphs were also constructed with time (t) as an explanatory variable to observe the impression about the variations and changes in trend (upward and downward) among the five climatic variables over the whole period (1989–2015).

**Figure 4:**
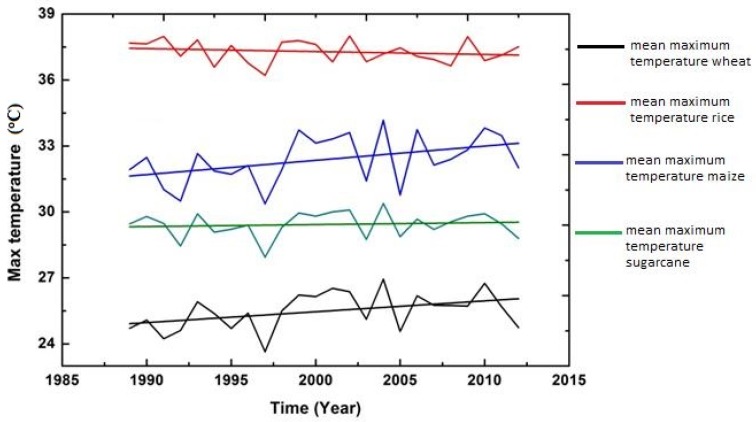
Trend of mean maximum temperature for Pakistan, 1989–2015.

**Figure 5:**
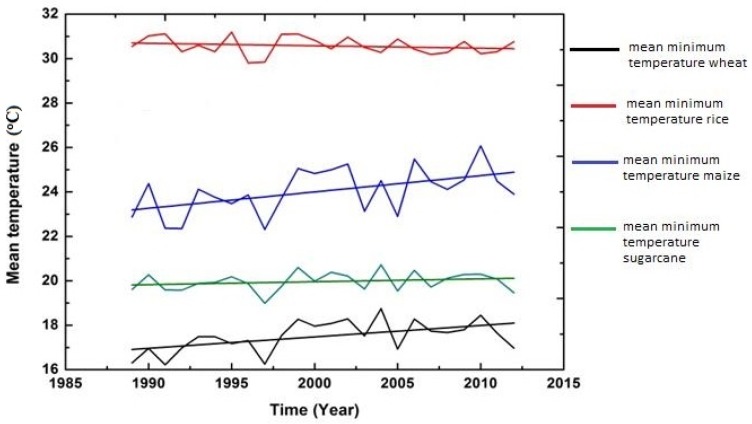
Trend of mean minimum temperature for Pakistan, 1989–2015.

**Figure 6:**
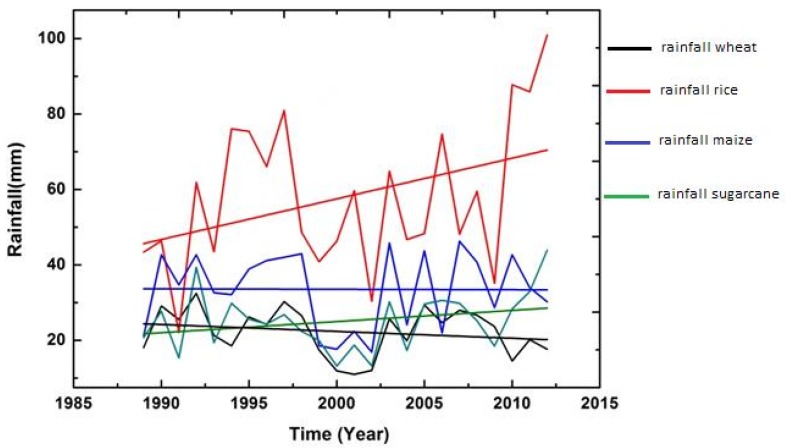
Trend of rainfall for Pakistan, 1989–2015.

**Figure 7:**
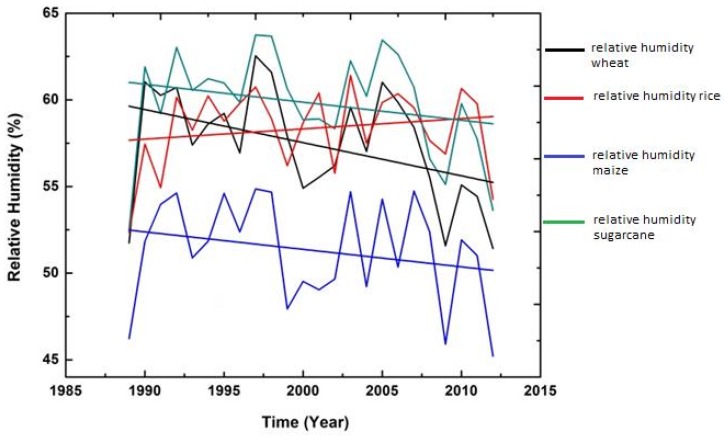
Trend of relative humidity for Pakistan, 1989–2015.

**Figure 8:**
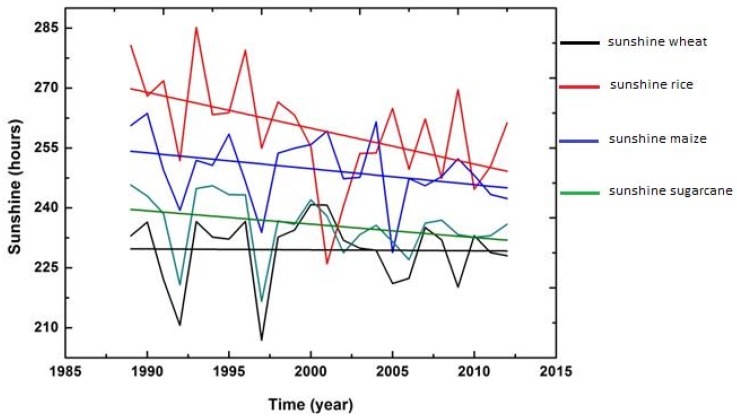
Trend of sunshine for Pakistan, 1989–2015.

The figures of all climatic variables are given in Figure 4, Figure 5, Figure 6, Figure 7 and Figure 8. The mean maximum temperature fluctuated slightly, and small variations were noticed in maximum temperature for all crops (Figure 4). However, the minimum temperature in rice and sugarcane growing season showed upward trends with distinct fluctuations (Figure 5). The trend in minimum temperature appears to be increasing. Rainfall fluctuated greatly, especially in rice growing season, and trends show an increasing rate. During the wheat and maize growing seasons, rainfall showed a slight decrease in overall trend, while rainfall also showed slight variations in the sugarcane growing season (Figure 6). The trend in relative humidity for all the selected crops appeared to be decreasing slightly, and the overall trend greatly fluctuated in the maize growing season (Figure 7). Interestingly, the sunshine for rice and maize crops exhibited a decreasing trend for all seasons, with slight fluctuations for all the crops (Figure 8).

#### 3.2.1. Climate and Wheat Crop

Wheat is the leading staple food crop of Pakistan, and hence the food security policy focuses mainly on the production of wheat. Wheat is deemed important due to its extensive use as a food and a relatively cheaper source for animal feed. The sowing period for wheat is winter and it is harvested in summer. Low temperature is conducive for the growth of wheat, while high temperature can cause a delay in seedling growth. Similarly, rainfall pattern causes damage to the production of wheat at harvesting time, ultimately leading to a situation of food insecurity in the country [15]. A decrease of 1.9% was observed in wheat production from 25.979 million tonnes to 25.478 million tonnes during the period from 2013–2014 to 2014–2015.

The main reasons for the decreased production were prolonged winter season and irregular rainfall [89]. The HAC method was used to identify the impacts of climate change on the yield of the wheat crop. The findings of the study are shown in Table 2. Maximum temperature showed a significant and negative influence on yield. Minimum temperature also showed a statistically significant influence, and the contribution was also found to be positive on yield. In the case of rainfall, it showed a non-significant influence and a negative contribution to the yield of wheat. Both the relative humidity and the sunshine expressed or showed non-significant influence, while relative humidity displayed negative effects. The sunshine showed a positive effect on yield. The adjusted *R*^2^ value implied that almost 30% of the yield variation of the wheat crop is influenced by the climatic variability. It was found that changes in temperature can significantly affect the production of wheat [90,91].

#### 3.2.2. Climate and Rice Crop

Rice is the second-largest staple food crop and exportable item for Pakistan. From the 2014–2015 fiscal years, rice export earned US$1.53 billion for Pakistan. There was a 3% recorded increase in the growth of rice over the period of 2013–2014 to 2014–2015 [89].

To determine the influence of the climatic variables on the rice crop yield, the FGLS method was employed. The results are presented in Table 2, which showed that the effects of all the climatic parameters except the maximum temperature were observed to be significant for the yield of the rice crop. Minimum temperature caused a negative effect and non-significant influence on the yield of the rice crop.

Both rainfall and sunshine also negatively contributed to the yield of this crop and were non-significant. Relative humidity contributed positively, but it showed a non-significant influence on rice yield. However, the adjusted *R*^2^ value showed that 33% variability in the yield of the rice crop is explained by climatic factors.

A study was conducted in which it was found that climatic variables significantly affect the yield of rice, but vary among different rice crops. Moreover, the effects of maximum temperature and minimum temperature are pronounced as compared to rainfall [92]. It is confirmed that rising maximum mean temperature would result in an increase in rice production, while the increase in minimum temperature would lead to an enhancement in the production [93]. Rice production trends could be estimated or expected by the use of the Autoregressive Integrated Moving Average (ARIMA) model, but it did not reflect climate influence [94]. Other studies also stated that an increase in temperature and rainfall negatively affects rice production [16,95,96].

#### 3.2.3. Climate and Maize Crop

Maize is an important food crop and a raw material for many products. The production of maize decreased by five percent from 4.6944 million tonnes to 4.695 million tonnes during the period 2013–2014 to 2014–2015 [89].The HAC method was performed to determine the climate change and maize yield relationship, and the findings are presented in Table 2. Results revealed that both maximum temperature and minimum temperature were found to be positive and non-significant. Relative humidity showed a statistically significant contribution to the yield of maize crop, but it negatively influenced the yield of crops. Both rainfall and sunshine showed no statistically significant contribution, and negatively influenced the yield of the maize crop. The adjusted R² value indicated that about 39% of the variation in the maize crop yield is explained by the climatic parameters.

#### 3.2.4. Climate and Sugarcane Crop

Sugarcane is one of the important cash crops of Pakistan. It earned US$171.78 million of precious foreign exchange during the period 2014–2015. The production of sugarcane reduced from 67.5 million to 62.5 million during 2014 to 2015 [89].

Sugarcane is planted in Pakistan in autumn and spring seasons, mostly under rainfed conditions. FGLS method was used to obtain the contribution of the climatic variables to the yield of sugarcane. The results indicated that maximum temperature is statistically significant and has a positive influence on the crop yield. Both minimum temperature and relative humidity showed a statistically significant relation and positively influenced the yield. Rainfall and sunshine both showed a negative influence on sugarcane crop yield. Maximum temperature, minimum temperature, and relative humidity all showed a statistically significant relationship.

## 4. Conclusions

Climate change is a global environmental threat to all economic sectors—specifically the agricultural sector. Pakistan has faced extreme weather events like untimely and heavy rainfall and flash floods in mountainous areas affecting huge damage to the major crops and properties of farmers. It is expected that the above-mentioned situation will increase as a function of climate change. Paying attention to the significance of agriculture to the country’s economy and rural livelihoods, the importance of climate change adaptation approaches is critical. Even though the adaptation strategies are very important, all farmers do not use such strategies. The majority of rural households and connected urban populations in developing countries as well as in Pakistan are highly dependent on agriculture. Therefore, adaptation to the negative impacts due to climate variability may be essential to encourage food security for the country and to protect the subsistence of rural households.

The prime aim of this study was to analyze the impact of climate change (e.g., maximum temperature, minimum temperature, rainfall, relative humidity, and sunshine) on major food crops of Pakistan, including wheat, rice, maize, and the cash crop sugarcane. The HAC and FGLS methods were employed to achieve the objectives and obtained mixed results. Some climate variables affect the crop yield negatively and significantly, while others are not significant. The most influential climatic variables for wheat crop production in Pakistan were observed to be maximum temperature, rainfall, and relative humidity. The finding confirmed that maximum temperature is significant and negatively influenced the yield of wheat crop, while rainfall and relative humidity are both insignificant and negatively influenced wheat crop yield. The influence of maximum temperature is significant for the rice crop. Both temperature and relative humidity displayed positive interrelation with sugarcane crop yield. Overall, climate change has adverse impacts on the yield of major food crops. Almost 60 percent of the Pakistani population is living below the poverty line. Moreover, the population is growing rapidly and the country will face the problem of food security in the near future. The government needs to take firm action to overcome this problem and ensure sufficient food for the masses.

## 5. Limitations and Future Research Direction

The national level data might not depict the true picture of the impact of climate change on different agroecological zones. Therefore, to take the regional differences into account, region-specific studies should be conducted. We know that Pakistan is a diverse and rich land, and in this way the discrepancies would be removed, resulting in a balanced picture of the country.

## Figures and Tables

**Table 1:** Descriptive statistics, 1989–2015.

Variables	Major Crops	Statistics					
		Mean	Std. Dev.	Min.	Max.	Skewness	Kurtosis
	Wheat	93.74	80.16	2.10	324.50	1.13	3.30
Cropping Area (hectare)	Rice	10.63	12.04	0.00	43.70	1.07	2.87
	Maize	15.89	16.70	0.90	52.20	0.99	2.48
	Sugarcane	20.63	34.18	0.00	126.40	1.78	4.74
	Wheat	210.21	209.57	2.90	901.70	1.53	4.58
Production (tons)	Rice	17.21	18.13	0.00	83.40	1.06	3.41
	Maize	27.44	27.07	0.80	137.10	1.54	5.67
	Sugarcane	1018.16	1707.40	0.00	6403.80	1.75	4.64
	Wheat	879.91	262.46	252.53	1912.22	0.53	3.82
Yield (kg/acre)	Rice	737.29	237.73	303.52	1315.23	0.40	2.27
	Maize	949.31	1038.17	287.11	9307.80	5.71	42.95
	Sugarcane	18,284.91	3603.22	8498.42	29,454.20	0.11	3.14
	Wheat	25.50	3.44	14.47	29.96	−1.90	5.93
Maximum	Rice	36.91	2.00	32.40	42.90	0.36	2.93
temperature (°C)	Maize	32.39	3.46	24.95	38.67	0.04	2.12
	Sugarcane	28.86	3.25	22.70	35.15	0.28	1.84
	Wheat	10.85	3.11	1.37	15.64	−1.62	5.10
Minimum	Rice	24.84	1.71	19.68	29.16	−0.34	3.38
temperature (°C)	Maize	18.29	3.87	9.93	28.42	0.34	2.14
	Sugarcane	14.81	3.17	8.86	21.48	0.09	2.10
	Wheat	22.28	17.58	0.00	71.18	0.79	2.75
Rainfall (mm/year)	Rice	70.89	60.02	0.00	322.58	1.31	4.58
	Maize	33.56	21.80	3.58	112.98	1.08	3.76
	Sugarcane	28.76	20.24	0.25	132.16	1.26	5.91
	Wheat	57.43	6.71	36.17	70.20	−0.74	3.40
Humidity (%)	Rice	58.57	5.90	44.80	74.00	0.09	2.82
	Maize	51.34	5.80	37.25	64.25	−0.21	2.36
	Sugarcane	60.35	5.87	43.55	73.43	−0.52	2.89
	Wheat	229.33	21.91	160.96	280.80	−0.24	3.33
Sunshine (h/day)	Rice	259.49	23.06	175.14	381.00	0.15	7.15
	Maize	249.24	18.37	190.35	290.35	−0.13	3.26
	Sugarcane	233.13	24.12	176.38	290.11	0.37	2.47

Source: Authors own estimation based on Pakistan Meteroloigcal Department (PMD, 2015).

**Table 2:** The regression results of different major crops of Pakistan evaluated by ordinary least square (OLS) and feasible generalized least square (FGLS).

Variables	Major Crops			
	Wheat	Rice	Maize	Sugarcane
Max Temp	−1.7991 *	3.9200 *	0.1174	0.4743 *
Min Temp	0.6216 *	−0.7041 *	0.5458	0.2578 *
Rainfall	−0.1195 *	−0.0126	−0.703	−0.0094
R.Humidity	−0.1107	0.183	−1.3219 *	0.3135 *
Sunshine	0.2169	−0.2114	−0.8761	−0.1253
Trend	0.0135 *	0.01447 *	0.0391 *	0.009 *
constant	4.5121 *	−11.0511 *	8.4222	0.8063
*F*	18.68 *	18.26 *	25.19 *	10.69 *
Wald chi^2^	96.53 *	87.75 *	101.09 *	60.69 *
*R*^2^	0.2993	0.3302	0.3932	0.2501
*n*	226	178	156	182

* represents level of statistical significance.
